# Interventions to facilitate interprofessional collaboration in the operating theatre: A scoping review

**DOI:** 10.1177/17504589221137978

**Published:** 2022-12-05

**Authors:** Marie-Julie Levesque, Cole Etherington, Michelle Lalonde, Narges Moradi, Lindsey Sikora, Dawn Stacey

**Affiliations:** 1 Faculty of Health Sciences, University of Ottawa, Ottawa, ON, Canada; 2 Ottawa Hospital Research Institute, Ottawa, ON, Canada; 3 Health Sciences Library, University of Ottawa, Ottawa, ON, Canada

**Keywords:** Interprofessional, Collaboration, Teamwork, Operating theatre, Nursing

## Abstract

**Background:**

Ineffective collaboration can increase adverse events in the operating theatre. When professionals work collaboratively, they are more likely to improve patient safety and outcomes.

**Aim:**

To identify interprofessional collaboration interventions involving operating theatre teams and describe their effect on facilitating communication, teamwork, and safety.

**Methods:**

A scoping review of four databases. Results were analysed by identifying interventions and mapping their related outcomes.

**Results:**

Twenty studies evaluated single or multi-faceted interventions. Despite low-quality study designs (no randomised controlled trials), four interventions (eg: briefings, checklists, team training, debriefing) improved communication and teamwork, and enhanced safety outcomes. Only one study, using team training, reported that organisational level interventions (eg: Standard Operating Procedures, Lean quality improvement management system) improved teamwork and safety outcomes.

**Conclusion:**

Several studies reported interventions enhanced interprofessional collaboration within operating theatre teams. Although findings were in favour of improved communication and teamwork, more rigorous research is required.

## Introduction and literature review

Ineffective interprofessional collaboration can have adverse effects on health care especially in the operating theatre (Mazzocco et al 2009, Muller et al 2018, Schwendimann et al 2018, Zegers et al 2011). A large proportion of surgical adverse events are preventable, and their consequences are more severe compared to other types of adverse events in health care (Schwendimann et al 2018, Zegers et al 2011). Interprofessional collaboration (IPC) in the operating theatre (eg: communication, teamwork) improves respect among health care professionals and can lead to improved patient safety and outcomes (Muller et al 2018, Wauben et al 2011, WHO 2010).

IPC happens when multiple health care workers of different professions provide comprehensive services to patients, their families, and communities (WHO 2010). The Interprofessional Education for Collaborative Patient-centred Practice (IECPCP) framework is a structural model to facilitate and support the implementation of an approach to IECPCP across health care sectors (D’Amour et al 2008, D’Amour & Oandasan 2005). The IECPCP has 3 levels (micro, meso, and macro) with four interrelated core dimensions (internalisation, shared goals and vision, governance, and formalisation). IPC interventions can enhance IPC education and practice (Reeves et al 2011, 2018, WHO 2010, 2021).

An IPC intervention targets members of more than one health and/or social care profession with the explicit purpose of improving IPC (Zwarenstein et al 2009). A Cochrane systematic review of practice-based IPC interventions within health care conducted in five countries identified nine individual and cluster-randomised controlled trials evaluating four types of practice-based IPC interventions: (a) externally facilitated interprofessional activities, (b) interprofessional rounds, (c) interprofessional meetings, and (d) interprofessional checklists (Reeves et al 2017). Of nine studies, one conducted in the operating theatre evaluated a checklist based on a literature review of surgical practices and consensus of two surgeons, (Calland et al 2011). Findings showed no improvement on patient safety and little improvement on IPC (eg: situational awareness did not significantly differ) within the operating theatre teams (Calland et al 2011). More recent research reports that IPC can promote improved patient safety and outcomes, quality of work and work environment in the operating theatre (Holmes et al 2020). However, operating theatre nurses are known to rate IPC lower compared to other health care professionals within the IP team (Bowles et al 2016, Makary et al 2006, Muller et al 2018). Given this previous synthesis was limited to randomised controlled trials, and there is variability in findings based on the discipline reporting outcomes, there is little synthesised evidence on the use of interventions to improve IPC in the operating theatre.

The aim of this scoping review was to identify IPC interventions involving operating theatre interprofessional teams and describe their effect on facilitating communication within the IP team, teamwork, and safety outcomes.

## Methods

### Study design

A scoping review was guided by the Arksey and O’Malley (2005) framework. The five steps were (a) identifying research questions, (b) identifying relevant studies, (c) selecting relevant studies, (d) data charting, and (e) collating, summarising, and reporting of study results.

#### Identifying research questions

The research questions were: (a) What types of interventions are used to improve IPC in the operating theatre? (b) What IPC interventions are more likely to be adhered to by operating theatre health care professionals? and (c) What is the reported effect of IPC interventions in the operating theatre on communication, teamwork, and safety outcomes?

#### Selecting relevant studies

SPIDER (Sample, Phenomenon of Interest, Design, Evaluation, Research type) (Cooke et al 2012) (Table 1) was used to determine study eligibility. Given the extent of the literature, the focus of this study was on eligible quantitative studies that evaluated interventions to facilitate IPC within the operating theatre with teams that included nurses, surgeons, and anaesthetists.

**Table 1:** Inclusion and exclusion for criteria for search strategy tool SPIDER

SPIDER	Inclusion criteria	Exclusion criteria
Sample	Nurses, surgeons, and anaesthetists	Nurses only, physicians and anaesthetists only
Phenomenon of interest	Interprofessional collaboration /teamwork in the operating room	Non-operating theatre environment
Design	Studies evaluating interventions using randomised controlled trials, pre–post, observational, and interrupted time series	Thesis, dissertations, quality improvement articles, editorials, and conference abstracts
Evaluation	Outcomes to measure the effect of the IPC interventions	
Research type	Quantitative	Qualitative research (eg: ethnographic, phenomenological, grounded theory)
Language	All languages that can be translated	

IPC: interprofessional collaboration.

##### Information sources

Medline (via OVID), CINAHL (via EBSCOHost), PsycINFO (via OVID), and Embase (via OVID) were searched between 1 January 2005 to 6 November 2020. The start date of 2005 reflects the publication date for the IECPCP framework.

##### Search strategy

With the assistance of research librarian (LS), the research team designed the search strategy. Subject headings and keywords included: Interprofessional, Interprofessional relations, Interdisciplinary, Multidisciplinary, Collaboration, Teamwork, Operating room, Nursing. The searches were performed by MJL and validated by LS (Appendix A).

#### Selecting relevant studies

The search findings were uploaded into Covidence (2020). Title and abstract screening were conducted by two independent authors (MJL, NM). Titles and abstracts were excluded only if both reviewers agreed. Studies that appeared to meet the inclusion criteria were retrieved in full and assessed independently by two reviewers (MJL, NM, DS). Disagreements were resolved through discussion and consensus.

#### Data charting

Three authors independently pilot tested the data extraction sheet (DS, MJL, NM) with two studies and extracted data compared to ensure consistency. Guided by the Arksey and O’Malley (2005) framework, data extraction included (a) study characteristics, (b) intervention characteristics, (c) instruments, and (d) outcomes (eg: communication, teamwork, safety).

#### Collating, summarising, and reporting of study results

Extracted data were mapped against the research questions (Arksey & O’Malley 2005). The research team analysed findings to respond to the research questions with results summarised in tables and described narratively. Results are reported using the PRISMA-ScR checklist (Tricco et al 2018).

## Results

**Figure 1:**
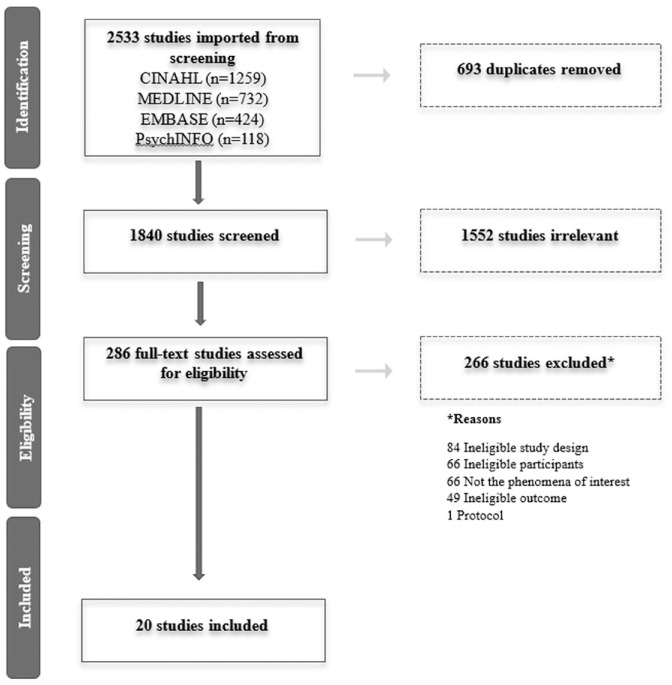
Selection of sources of evidence PRISMA flow diagram

Of 1840 citations after removing duplicates, 20 studies were eligible to be included. Reasons for excluded full text were documented. See the PRISMA flow diagram (Figure 1) for details (Tricco et al 2018).

### Characteristics of sources of evidence

Studies, conducted in eight countries; United States (n = 10), United Kingdom (n = 4), Brazil (n = 1), Canada (n = 1), France (n = 1), Finland (n = 1), Israel (n = 1), and New Zealand (n = 1) (Table 2), were mostly in academic hospitals (75%; n = 15). Studies were (a) non-randomised intervention with controlled group or interrupted time series (10%; n = 2) (McCulloch et al 2017, Morgan et al 2015), (b) pre–post using observations or survey (55%; n = 11), and (c) mixed method using observation and survey (35%; n = 7). Studies’ participants were members of the operating theatre team including: (a) surgeons and often residents, (b) anaesthetists and often residents, and (c) nurses (90%; n = 18). The other two studies did not describe the team members (Cabral et al 2016, Hinde et al 2016). The median number of participants who responded to surveys was 129.5 (range = 32–929) and median of 86 observations of surgical cases (range = 34–37,133) (see Table 2).

**Table 2:** Characteristics of included studies

Author, Year, Country	Setting (# Beds)	Study Aim	Study methods and timing	Interventions	Participants^a^
1. Ali et al (2011) UK	Two academic hospitals (1200)	To evaluate implementation of safety briefings and understand issues affecting this process of change.	Pre–post observations at baseline 2 months and postsurvey at 2 months	briefing, debriefing, and checklist/adaptation WHO SSC	Presurgery observed N = 27; Post N = 34 Survey N = 37 (n/r surgeons, anaesthetists, nurses)
2. Armour Forse et al (2011) USA	One academic hospital	To determine if team training improves operating theatre performance and culture.	Pre–post observations, pre–post survey at baseline, 1 year	Team training – Programme (TeamSTEPPS)	N = n/r entire surgical service (surgeons, anaesthetists, residents, nurses, scrub technicians)
3. Awad et al (2005) USA	One (n/r) hospital	To determine if communication in the operating theatre could be improved through medical team training.	Pre–post survey at baseline and 2 months	Team training (CRM) and briefing	N = n/r entire surgical service (nurses, anaesthetists, surgeons)
4. Berenholtz et al (2009) USA	One academic hospital (1061) (58 operating theatres)	Evaluate the effect of a briefing and debriefing tool on perceptions of interdisciplinary communication.	Observations from previous 2 years and postsurvey at 2 years	Briefing and debriefing	N = 8 operating theatre teams (37,133 briefing and debriefing) Survey N = 40 (10 surgeons, 10 anaesthetists, 20 nurses)
5. Cabral et al (2016) USA	One community hospital (204) (8 operating theatres)	To assess changes in relationships and postoperative outcomes after implementing modified WHO SSC.	Pre–post survey at baseline and 2 months	Checklist/adaptation WHO SSC and briefing and debriefing	N = 93 (19 surgeons, 33 nurses, 21 surgical technologists, 20 others) Pre N = 47 of 93 Post N = 46 of 93
6. Columbus et al (2018) USA	One academic hospital (43 operating theatres)	[To determine] if the use of an evidence-based communication tool aimed to trigger intraoperative discussion improve communication in the operating theatre.	Pre–post survey at baseline and 9 weeks	Checklist/ Adaptation WHO SSC	Pre N = 103 of 514 (42 surgeons, 38 anaesthetists, 23 nurses) Post N = 81 (22 surgeons, 31 anaesthetists, 28 nurses)
7. Einav (2010) Israel	One academic hospital	To develop a briefing protocol and evaluate its effect on patient safety.	Pre–post observations for 3 months at baseline and a year later and postsurvey at 3 months	Briefing	Surgeries observed N = 232 130 without and 102 with briefing Survey N = 32 (n/r surgeons, anaesthetists, nurses)
8. Gore et al (2010) USA	One academic hospital	To evaluate the perceived efficacy of crew resource management initiative on preop briefing in the operating theatre.	Pre–post survey at baseline and 6 months	Team training (CRM) and briefing	Pre N = 207of 600 (109 surgeons, 29 anaesthetists, 49 nurses, 20 others) Post N = 156 of 565 (65 surgeons, 17 anaesthetists, 46 nurses, 29 others)
9. Hacquard et al (2013) France	One academic hospital (27 operating theatres)	To assess the perception of medical and nursing staff regarding the advantages of the checklist and its level of integration within the overall organisation of the operating room.	Postobservations and postsurvey at 1 year	Checklist/adaptation WHO SSC	Surgeries observed N = 64 Survey N = 177 of 201 (36 % surgeons /residents, 16% anaesthetists, 43% nurses)
10. Halverson et al (2009) USA	One academic hospital	To develop and implement a team-training curriculum.	Pre–post observations and pre–post survey at baseline and 6 months	Team training (CRM) and briefing and debriefing	Presurgery N = 39 post N = 37 Surveys Pre–post N = 156 (95 nurses, 34 anaesthetists /residents, physicians, 27 surgeons /residents)
11. Hinde et al (2016) UK	One academic hospital	To assess the impact of interprofessional point of care simulation on the safety culture of operating theatres.	Pre–post survey at baseline and at 6 to 12 months	Training (in situ simulation)	N = 84 (45 nurses, 14 healthcare assistants, 15 operating theatre practitioners, 10 physicians) Survey pre and post N = 46 of 72 (n/r)
12. Lingard (2008) Canada	One academic hospital	To assess whether structured team briefings improve operating theatre communication.	Pre–post observations at baseline and 5 months and post survey at 5 months	Briefing	Surgical Observations N = 172 (86 pre and 86 post) Survey N = 77 of 83 (22 surgeons /residents, 23 nurses, 32 anaesthetists /residents)
13. Makary et al (2007) USA	One academic hospital	To evaluate the impact of operating theatre briefings on coordination of care and risk for wrong-site surgery.	Pre–post survey at baseline and 3 months	Briefing	N = 422 (34.9% surgeons /residents, 14% anaesthetists /residents 44.3% nurses 4.8 % medical nursing students 2% others) pre N = 306 of 360 post N = 116 of 154
14. McCulloch et al (2017) UK	Five hospitals	To compare improvement in surgical team performance after interventions addressing teamwork culture, work systems, or both.	Pre–post observations comparing nonrandomised intervention group and control group at baseline and 4 months	Team training (CRM) and systems redesign and standardisation (SOP) and Lean quality improvement	N = 453 operations (255 intervention, 198 control). (surgeons, nurses, anaesthetists, and others)
15. Molina et al (2016) USA	Thirteen hospitals	To measure perception of multiple dimensions of perioperative safety among clinical operating theatre personnel before and after implementation of an SSC.	Pre–post survey at baseline and at 1 to 2 years	Checklist/adaptation WHO SSC	Pre N = 929 of 1921 Post N = 815 of 1909 (198 surgeons, 42 anaesthetists, 436 nurses, 161 surgical technicians, 81 others, 11 missing)
16. Morgan et al (2015) UK	One District general hospital	To evaluate the effectiveness of aviation style teamwork training in improving operating theatre team performance and clinical outcomes.	Pre–post Observations using interrupting time series comparing non-randomised intervention group and control group at baseline and 3 months	Team training (CRM) and WHO SSC	Surgical case observations N = 72 operations; 37 intervention, 35 control. 3 operating theatre sub teams (anaesthesia, surgery, nursing)
17. Nundy (2008) USA	One academic hospital	To evaluate the impact of briefings on operative delays.	Pre–post survey at baseline and at 3 to 5 months	Briefing	N = 422 (147 surgeons /residents, 59 anaesthetists /residents, 187 nurses, 6 nurse assistants or PA, 16 medical students, 7 others) Pre N = 306 of 360 Post N = 116 of 154
18. Ong et al (2016) New Zealand	One community hospital	To evaluate engagement of operating theatre sub teams and compliance with administering checklist domains and items, after introducing a wall mounted paperless checklist with migration of process leadership.	Pre–post observations at baseline with WHO SSC and 2 year later with wall poster 2 months postimplementation	Checklist/adaptation WHO SSC wall poster without documentation	Surgical Observations N = 111 3 operating theatre sub teams (anaesthesia, surgery, nursing)
19. Santana et al (2016) Brazil	Three academics hospitals	To evaluate the attitudes and opinions regarding surgical safety among operating theatre professionals in these hospitals before and after implementation of the checklist.	Pre–post survey at baseline and 2 years	Checklist/WHO SSC	N = 472 (141 surgeons /residents, 90 anaesthetists /residents, 57 nurses, 123 nursing technicians, 45 nurses assistants, 46 others) Pre N = 257 of 472 post N = 215 of 472
20. Takala et al (2011) Finland	Four academics hospitals	To assess the impact on the operating theatre process, safety-related issues, and communication among surgical staff in a high-income country.	Pre–post survey at baseline and at 2 to 4 weeks	Checklist/adaptation WHO SSC	Pre N = 901 post N = 847 of 1748 operations (n/r surgeons anaesthetists, nurses)

SOP: standard operating procedure; WHO: World Health Organization; SSC: Surgical Safety Checklist; CRM: crew resource management; TeamSTEPPS: Team Strategies and Tools to Enhance Performance and Patient Safety.

a Participants: nurses may include certified registered anaesthetic nurses, scrub and circulating nurses.

### Characteristics of interventions to improve IPC in the operating theatre and their use

Four IPC interventions evaluated were (a) briefings (50%; n = 10 studies), (b) checklists (45%; n = 9), (c) team training (35%; n = 7), and (d) debriefings (20%; n = 4). Studies evaluated an individual intervention (50%, n = 10) or a multi-faceted intervention (50%, n = 10) (Table 3).

**Table 3:** Outcomes for IPC interventions

Intervention	Author, Year	Outcomes involving nurses		
		Communication	Teamwork	Safety outcomes
Checklist	Columbus et al (2018)	87.34% improved team communication Unchanged speaking out behaviour with training (p = 0.257). Surgeons empowered to speak up (p = 0.05) not significant for nurses (p = 0.65) and anaesthetists (p = 0.70).	Increase in perceived bravery (p = 0.049). Likelihood to take control (p = 0.05).	Improve awareness of patient disposition but not significant for surgeons (p = 0.10) and anaesthetists (p = 0.84). Nurses reported being less aware of change in patient disposition (p = 0.22). Decreased awareness of intraoperative blood product transfusion during cases but not significant (p = 0.51). Increase awareness of intraoperative blood product transfusion during cases but not significant for surgeons (p = 0.31) and anaesthetists (p = 0.55).
	Hacquard et al (2013)	40% disagree that the checklist improves communication.	n/r	33% detected an error with the checklist.
	Molina et al (2016)	11.9% relative improvement (p < 0.05).	Improved all 5 teamwork dimensions (3.6% respect, 3.5% clinical leadership, 5.7% assertiveness, 2.9 % coordination, 11.9% communication; p < 0.05).	73.6% averted problems or complications.
	Ong et al (2016)	n/r	n/r	n/r
	Santana et al (2016)	92.7% improved for nurses, 87.9% anaesthetists, 75.6% surgeons, with significant diff (p < 0.001).	Improvement perception of safety and agreement about the collaboration of the operating team, for nurses (p = 0.001) anaesthetists (p = 0.046) not significant for surgeons (p = 0.50). Improved teamwork nurses (p < 0.001) anaesthetists (p = 0.038) not significant for surgeons (p = 0.49).	Improved concerns about patient safety and compliance with standards, rules, and hand-washing practices post-intervention, by anaesthetists, surgeons, and nurses (p < 0.001). 90.0% agreed checklist helps prevent errors.
	Takala et al (2011)	Improved discussions of critical events preoperatively (anaesthetists: 22.0% vs. 42.6%, Surgeons: 34.7% vs. 46.2%; p < 0.001). Fewer communication failures (43 vs. 17; p < 0.05).	Improved knowledge of names and roles among team members (anaesthetists: 65.7% vs. 81.8%, Surgeons: 71.1% vs. 83.6% Nurses: 87.7% vs. 93.2%; p < 0.01).	Patient’s identity was more often confirmed (anaesthetists: 62.7% vs. 84.0%, Surgeons: 71.6% vs. 85.5%, Nurses: 81.6% vs. 94.2%; p < 0.001).
Briefing	Einav (2010)	n/r	n/r	25% reduction in the number of nonroutine events when briefing was conducted. 16% increase in the number of surgeries performed without any nonroutine event. 5% and 11% reduction in the number of surgeries with one to two and three or more events (p < 0.02).
	Lingard (2008)	Mean communication failures per procedure declined (p < 0.001).	92% agreed that the briefing allowed the team to identify and resolve problems.	88% agreed briefing helped guard against mistakes. 33% agreed briefings showed utility: identify problems, resolution of critical knowledge gaps, decision-making, follow-up actions.
	Makary et al (2007)	Agreed team discussions are common (pre–post 52.4%-64.4%; p < 0.001).	Briefings perceived to improved collaboration (p < 0.001). Agreed decision making utilised input from relevant personnel (pre–post 78.7%–89.6%; p < 0.003). Surgeons and anaesthetists worked together as a well-coordinated team” (pre–post 67.9%–91.5%; p < 0.000).	Briefings perceived to reduced risk for wrong-site surgery (p < 0.001). Agreed preoperative briefing increased aware of surgical site (pre–post 52.4%–64.4%; p < 0.001). Agreed surgical site was clear to me before incision (pre–post 88.2%–96.6%; p < 0.002). Briefings associated with caregiver perceptions of reduced risk for wrong-site surgery (p < 0.001). A team discussion before a surgical. procedure is important for patient safety (pre–post 94.0%–93.3%; p < 0.123).
	Nundy (2008)	19% fewer communication breakdowns leading to delays (p < 0.006).	n/r	31% reduction in unexpected delays. 36% reported delays pre and 25% reported delays in the postintervention period (p < 0.04).
Team training	Armour Forse et al (2011)	Improved communication (p < 0.05).	Improved teamwork (p < 0.05).	Improvement in first case starts (69% to 81%) A year later decreased (81% to 69%; p < 0.05). Patient satisfaction improved. (77% to 89.3%; p < 0.05) A year later decreased to 80.8%. Improved antibiotics use (78%–97%; p < 0.05). Improved anti-venous thromboembolism use (74% –91%; p < 0.05). Improved Beta blocker use (19.7%–100%; p < 0.05). Decreased mortality (2.7%–1.0%; p < 0.05) and 1 year later increased 1.5%. Decreased morbidity (20.2%–11.0%; p < 0.05) and 1 year later increased to 13%.
	Hinde et al (2016)	n/r	Improved teamwork (p = 0.013).	90% increased awareness of critical incidents. 85% increased confidence in dealing with critical incidents. Improved safety (p < 0.001).
Team training + SOP + Lean	McCulloch et al (2017)	n/r	NOTECHS II score rose post intervention in the pooled active groups (72.98 pre, 76.56 post) but not in the control groups (73.31 pre, 73.03 post) Improvement (p < 0.025).	Mean glitch rate in active groups decreased, in the control group it rose (p = 0.0014).
Briefing + Team training	Awad et al (2005)	Improved communication for surgeon (p < 0.0004); and anaesthetists (p < 0.0008); no diff for nurses (p = 0.7).	n/r	Pre–post 84%–95% prophylactic antibiotics use within 60 min .Pre-post 92%–100% sequential compression devices prior to induction .3.3% (7/213) patients identified before induction at high risk for surgery.
	Gore et al (2010)	n/r	3/4 questions (75%) improved perception of teamwork for nurses (no diff for residents, physicians).	3/11 questions (27%) improved patient safety for nurses (no diff for residents, physicians) .1/13 questions (8%) improved error reporting for nurses and residents (no diff physicians).
Briefing + Debriefing	Berenholtz et al (2009)	90% agreed for briefings 69% for debriefings.	90% agreed for briefing; 72% for debriefings.	n/r
Team training + Checklist	Morgan et al (2015)	n/r	Mean NOTECHS II score increased from 71.6 to 75.4 in the active group but remained static in the control group (p = 0.047). NOTECHS II nursing score increased (p = 0.006), but the anaesthetic and surgical scores did not.	Mean glitch rate was unchanged in the control group but increased significantly (7.2–10.2/h, p = 0.002) in active group. Rise in the complication rate in the active group after the intervention and a fall in the rate in the control group (p = 0.05).
Briefing, + Debriefing + Checklist	Ali et al (2011)	89% improved communication.	n/r	89% more aware of cases .97% highlighted potential patient problems.
	Cabral et al (2016)	6% increased communication (p < 0.05) 12% increased nurses’ perception of communication (p = 0.002).	Decreased perception of teamwork (p = 0.29).	Decreased Safety Climate (p = 0.48).
Briefing + Debriefing + Team training	Halverson et al (2009)	72% increased tension owing to information that could have been communicated during a briefing.	Improved perception of teamwork in 14 out 19 questions. 75% greater sense of teamwork with briefing (nurses vs anaesthetists, p = 0.13; nurses vs surgeons, p < 0.001).	37% communicated information during the briefing that if not communicated would have led to an increased risk for the patient or a delay in the case.

*n/r*: not reported; diff: difference; NOTECHS II: Non-Technical Skills updated version; P: P value; SOP: Standard Operating Procedures.

#### Briefings

Ten studies evaluated a briefing intervention (Table 3). A briefing refers to a communication practice among the surgical team members before the surgical procedure to help enhance knowledge, purposeful action, quality, and safety of collaborative care (eg: team briefing, perioperative briefing). Briefings were applied in various formats (eg: list of structured items or poster) with some documented and others verbal only.

#### Checklists

Nine studies evaluated the WHO Surgical Safety Checklist (SSC) or a version adapted for their setting (Table 3). The WHO SSC is a 19-item checklist where the entire team stops at three critical points: (a) pre-anaesthesia (Sign In), (b) pre-incision (Time Out), and (c) before patient leaves the operating theatre (Sign Out) to enhance communication between the surgical team members, improve outcomes, decrease complications, and improve patient safety (WHO 2009). The WHO SSC has a designated area of responsibility for each of the three operating theatre sub teams (eg: surgery, anaesthesia, nursing). Nurses frequently contributed to the initiation and systematic verification of the checklist, but the checklist is not always used completely (Hacquard et al 2013, Molina et al 2016). The SSC significantly improved Sign Out compliance and team engagement by all operating theatre sub teams (Ong et al 2016).

#### Team training

Seven studies used a variety of team training interventions (Table 3). Team training in health care refers to an educational programme provided to a group of health care professionals to build overall team performance by increasing their procedural knowledge, proficiency in their roles, and skills in functioning as part of a team. Of seven studies, five used the Crew Resource Management (CRM) approach (Awad et al 2005, Gore et al 2010, Halverson et al 2009, McCulloch et al 2017, Morgan et al 2015). This approach is based on the aviation-style CRM communication techniques and consists of a body of basic patient safety behaviours, including leadership, assertiveness with respect, and effective communication techniques (Awad et al 2005, Gore et al 2010). One study used CRM with systematic multi-organisational level team training approaches such as Standard Operating Procedures (SOP) and the Lean quality improvement management system (Lean) (McCulloch et al 2017). Another study used the TeamSTEPPS programme (Armour Forse et al 2011); a government sponsored programme composed of 12 modules that provides a rich resource-based, evidence-based approach for training groups of health professionals to improve institutional collaboration and communication relating to patient safety (Canadian Patient Safety Institute 2021). The final study used point of care (in situ) simulation on safety culture in the operating theatre (Hinde et al 2016). Most studies used team training as a multi-faceted intervention (Table 3). Team training was described as significantly improving compliance with briefings and debriefings (Halverson et al 2009).

#### Debriefings

Four studies specifically mentioned the use of debriefing as part of a multi-faceted intervention (Table 3). Debriefing refers to a structured communication among the surgical team members after the surgical procedure to review any concerns or deficits identified during the procedure. Some studies refer to debriefing as a ‘sign-out’ from a surgical safety checklist. A high percentage (70%) of the operating theatre team members agreed that the debriefing tool is feasible considering their workload (Berenholtz et al 2009).

### Reported effects of IPC interventions on communication, teamwork, and safety outcomes

The IPC interventions are presented as a single or a multi-faceted intervention and in order of frequency (Table 3).

#### Single-interventions

##### Checklist intervention (*n* = 6 studies)

Five of six studies used an adapted version of the WHO SCC and one study using the original WHO SSC (Table 3). Four out of six studies that measured team communication reported improved team communication. Although one study reported improved team communication, the authors also reported that the checklist did not improve the ability to raise patient safety concerns (ie: ‘speaking out behaviour’) by the nurses and anaesthetists (Columbus et al 2018). All four studies that measured teamwork reported improvements as indicated by enhanced assertiveness, respect, clinical leadership, coordination, and collaboration (Columbus et al 2018, Molina et al 2016, Santana et al 2016, Takala et al 2011). Of the five studies that measured safety outcomes, four reported the checklist helped prevent errors and improved compliance with standards of practices (Hacquard et al 2013, Molina et al 2016, Santana et al 2016, Takala et al 2011) and one study showed no differences (Columbus et al 2018).

##### Briefing intervention (n = 4)

Three of four studies that measured communication reported significant improvements by reducing communication failures, increasing teamwork discussions, or reducing communication breakdown leading to delays (Table 3). For two studies, teamwork was significantly enhanced by improving the perception of collaboration, decision making used, or allowing the team to identify and resolve problems. Patient safety was improved in all four studies by either reducing non-routine events (eg: near misses), resolving critical knowledge gaps, helping prevent mistakes, augmenting surgical awareness, and reducing risk of wrong-site surgery.

##### Team training intervention (n = 3)

Of three studies, one measured and reported significant improvement in communication following the TeamSTEPPS programme (Table 3). All three studies showed improvements in teamwork and in safety outcomes. For example, the study using simulation training revealed statistically significant perceived improvements in both teamwork (p = 0.013) and safety climate scores (p < 0.001) and improved awareness of and confidence in dealing with critical incidents six and 12 months after implementation of interprofessional point of care simulation sessions (Hinde et al 2016). The study that used the TeamSTEPPS programme showed increased use in antibiotics, beta blockers, and venous thromboembolism treatment with a decrease in mortality and morbidity rates and persistency one year after implementation (Armour Forse et al 2011). The final study showed decreased mean glitch rate compared to increased mean glitch rate for controls (McCulloch et al 2017).

#### Multi-faceted intervention

##### Briefing and team training (n = 2)

Two studies employed briefing and CRM team training (Table 3). One study measured and reported improved communication for surgeons and anaesthetists, but no difference for nurses (Awad et al 2005). The other study measured and reported improved teamwork for nurses, but no difference for other team members (Gore et al 2010). Both studies reported overall improvement in patient safety with nurses increasing reporting of patient safety and errors (Gore et al 2010) and increased application of preventive measures during operating theatre procedures (eg: prophylactic antibiotics within 60 minutes and sequential compression devices prior to induction) (Awad et al 2005).

##### Briefing and debriefing (n = 1)

One study used briefing and debriefing interventions (Berenholtz et al 2009). Both briefing and debriefing were perceived to improve communication and teamwork, but the study did not measure safety outcomes.

##### Team training and checklist (n = 1)

One study used CRM team training and the WHO SSC (Morgan et al 2015). No communication or safety outcomes were measured. There was improved teamwork, such as situation awareness, decision making, leadership and cooperation. Concurrently, there was a rise in operative glitches such as interruptions, omissions and changes affecting outcomes of the procedure.

##### Briefing, checklist, and debriefing (n = 2)

Two studies used briefing, debriefing and the WHO SSC (Table 3). There was improved communication and perception of communication in both studies. One study reported no differences in teamwork and safety outcomes (Cabral et al 2016). The other study reported improved safety outcomes by increased awareness during cases and enlightenment of potential problems (Ali et al 2011).

##### Briefing, debriefing, and team training (n = 1)

One study applied briefing, debriefing, and CRM team training (Halverson et al 2009). The communication between team members was improved with the use of briefings by helping alleviate operating theatre team tensions. There was improved perception of teamwork and nurses reported better teamwork, more predominantly when briefings were used. Patient safety was improved by increased information exchanges, lessening the risks for patients and delay of care.

## Discussion

This scoping review identified interventions for improving IPC in the operating theatre were briefings, checklists, team training, and debriefings. Most studies reported a significant improvement in communication, teamwork, and safety outcomes. Some of the studies reported that nurses indicated less improvements compared to surgeons, anaesthetists, and others on the operating theatre team, but these results were not significantly different (Awad et al 2005, Cabral et al 2016, Columbus et al 2018). Overall, the findings from this scoping review indicated favourable improvements in patient safety and outcomes. However, these results need to be considered within the context of weak study designs and the need for further rigorous mixed-methods studies (Reeves et al 2017). These findings lead to the following points of discussion.

There were different perceptions of positive outcomes among the various professions within the operating theatre team. For example, nurses rated communication and teamwork lower compared to surgeons and anaesthetists (Carney et al 2010). This is consistent with findings from previous research showing discrepancies between surgical team members concerning communication, teamwork, and situation awareness (Gillespie et al 2013, Wauben et al 2011). All team members should understand and be well informed about the surgical procedures and about patient-specific health issues such as allergies or comorbidities (Wauben et al 2011). A lack of consistent perceptions between surgical team members can translate into a lack of shared understanding, leading to increased adverse events (Haynes et al 2009, Sexton et al 2006).

Most studies reported outcomes immediately after exposure to the intervention. In fact, only six of the 20 studies measured outcomes one year and beyond (Armour Forse et al 2011, Berenholtz et al 2009, Hacquard et al 2013, Molina et al 2016, Ong et al 2016, Santana et al 2016) (Table 3). Although most studies were showing favourable improvements overall, one study showed that the improved communication and teamwork at one year was better than baseline, but not as strong as the earlier post intervention measurements (Armour Forse et al 2011). These findings reinforce the recommendation from the previous Cochrane systematic review, suggesting waiting a longer period of time (unspecified) after implementation of the intervention before evaluating outcomes (Reeves et al 2017).

The second most common intervention was the WHO SSC or an adapted version. The WHO SSC is used around the world and in Canada for enhancing organisational practices (Accreditation Canada 2020, Healthcare Excellence Canada 2021). The WHO SSC has shown significant reduction in morbidity and mortality (Haynes et al 2009). Interestingly, one study from Canada, reported mixed findings on the effectiveness of the WHO SSC, and found that the SSC was not associated with significant reductions in operative mortality or complications (Urbach et al 2014). Urbach et al (2014), question if the favourable effect indicates a Hawthorne Effect. This explication is consistent with findings of a recent Systemic Review of the Hawthorne Effect on surgical studies, showing that 63% of the 16 included studies used this effect to explain their improvements in results (Demetriou et al 2019).

The least used intervention was debriefing, and it was always used in combination with one or more other interventions (eg: checklist, briefing, team training). The interchangeability of terminology of ‘debriefing’ and ‘sign-out’ of the surgical safety checklist in the literature may add confusion on the concept of debriefing. Hence, it is difficult to know the added effect of debriefing. The term ‘debriefing’ should refer to the additional communication (beyond acknowledgement of the tasks performed) at the end of the surgical case addressing safety, equipment and efficiency that arose, and identify opportunity for improvement (Brindle et al 2018). Compared to briefing at the start of operating theatre procedures, debriefing was shown to have less impact on communication and teamwork (Berenholtz et al 2009). Debriefing was less well implemented or accepted by IPC teams, and could be attributable to logistical challenges (eg: competing priorities of subsequent surgeries) and perceived lack of value (eg: not related to direct patient care, not valued by the institution, issues during debriefings not subsequently addressed) (Bergs et al 2015). To ensure meaningful debriefing, an atmosphere dedicated to open communication needs to be fostered with the commitment of resources (institutional and personnel) and leadership engagement (Brindle et al 2018).

Most team training within the operating theatre setting used CRM and identified that its application required more than just the micro IP operating theatre team involvement. For example, one study reported that when synthesising team training approaches at various levels (micro: CRM in operating theatre; meso: Lean and SOP adoption by the organisation), the effectiveness of the interventions to enhance safety was improved (McCulloch et al 2017). All the interventions targeted mostly the interactional processes of the IECPCP Framework (D’Amour & Oandasan 2005). It is important to recognise that collaboration does not only exist within the operating theatre team, but also in the context of a larger organisational setting within all dimensions of IPC (sense of belonging, shared goals, structure of care and governance) (D’Amour & Oandasan 2005). Therefore, strategies to improve IPC should consider interventions targeting micro (eg: operating theatre team), meso (eg: hospital policies and processes), and macro (eg: government and professional guidance). Furthermore, the IPC interventions should target organisation factors and systemic determinants (eg: strong leadership, human resource management, policies and governance supporting IPC) (D’Amour & Oandasan 2005, Reeves et al 2017, San Martin-Rodriguez et al 2005, Toh et al 2017).

## Limitations of the study

There are four key limitations that need to be considered. First, out of 20 included studies, 18 used weak study designs such as pre–post observations and/or survey evaluations. Second, there is potential for self-reported bias. To overcome the potential for self-response bias, several studies used observations to measure outcomes such as compliance, non-routine events, glitch rates, and communication and teamwork interactions. Third, assessment of methodological quality within this scoping review was not performed. This is common practice for scoping reviews unless there is a specific requirement due to the nature of the aim of the scoping review (Munn et al 2018). Fourth, there is a need to consider the potential impact of the Hawthorne effect also referred as the ‘observer effect,’ in which participants change their behaviour when being observed (Nguyen et al 2018). Eight out of ten studies described the Hawthorne effect as an explanation for the improvements in outcomes in surgical studies (Demetriou et al 2019).

## Conclusions and suggestions for further research

In the operating theatre, four IPC interventions have been evaluated in studies to measure their effect on enhancing communication, teamwork, and safety outcomes. The findings of this scoping review have focused mainly on the interpersonal processes for implementation of briefings, checklists, team training and debriefings. The included studies had low-quality designs and their outcomes reported improved IPC in the operating theatre. Therefore, there is not sufficient evidence to draw clear conclusions on the effects of IPC interventions in the operating theatres and more rigorous research using high-quality study designs is needed. Interestingly organisational factors influencing IPC were not reported, and it would be beneficial to better understand how the factors within the meso and macro levels influence the micro level IPC and outcomes. Other studies highlight the need to further improve the effectiveness of IPC multi-faceted interventions targeting individuals (eg: nurses, surgeons, anaesthetists), and systems-level factors within health care delivery (D’Amour & Oandasan 2005, Etherington et al 2021, Reeves et al 2017).

## Supplemental Material

sj-docx-1-ppj-10.1177_17504589221137978 – Supplemental material for Interventions to facilitate interprofessional collaboration in the operating theatre: A scoping review Click here for additional data file. Supplemental material, sj-docx-1-ppj-10.1177_17504589221137978 for Interventions to facilitate interprofessional collaboration in the operating theatre: A scoping review by Marie-Julie Levesque, Cole Etherington, Michelle Lalonde, Narges Moradi, Lindsey Sikora and Dawn Stacey in Journal of Perioperative Practice

## References

[bibr1-17504589221137978] Accreditation Canada 2020 Required Organizational Practices 2020 Handbook Ottawa, ON, Canada, Accreditation Canada

[bibr2-17504589221137978] Ali M Osborne A Bethune R Pullyblank A 2011 Preoperative surgical briefings do not delay operating room start times and are popular with surgical team members Journal of Patient Safety 7(3) 139–143 21857241 10.1097/PTS.0b013e31822a9fbc

[bibr3-17504589221137978] Arksey H O’Malley L 2005 Scoping studies: Towards a methodological framework International Journal of Social Research Methodology 8(1) 19–32

[bibr4-17504589221137978] Armour Forse R Bramble JD McQuillan R 2011 Team training can improve operating room performance Surgery 150(4) 771–778 22000190 10.1016/j.surg.2011.07.076

[bibr5-17504589221137978] Awad SS Fagan SP Bellows C , et al 2005 Bridging the communication gap in the operating room with medical team training American Journal of Surgery 190(5) 770–774 16226956 10.1016/j.amjsurg.2005.07.018

[bibr6-17504589221137978] Berenholtz SM Schumacher K Hayanga AJ , et al 2009 Implementing standardized operating room briefings and debriefings at a large regional medical center Joint Commission Journal on Quality and Patient Safety 35(8) 391–397 19719074 10.1016/s1553-7250(09)35055-2

[bibr7-17504589221137978] Bergs J Lambrechts F Simons P , et al 2015 Barriers and facilitators related to the implementation of surgical safety checklists: A systematic review of the qualitative evidence BMJ Quality & Safety 24(12) 776–786 10.1136/bmjqs-2015-004021 26199428

[bibr8-17504589221137978] Bowles D McIntosh G Hemrajani R , et al 2016 Nurse-physician collaboration in an academic medical centre: The influence of organisational and individual factors Journal of Interprofessional Care 30(5) 655–660 27388560 10.1080/13561820.2016.1201464

[bibr9-17504589221137978] Brindle ME Henrich N Foster A , et al 2018 Implementation of surgical debriefing programs in large health systems: An exploratory qualitative analysis BMC Health Services Research 18(1) 210 29580254 10.1186/s12913-018-3003-3 PMC5870386

[bibr10-17504589221137978] Cabral RA Eggenberger T Keller K Gallison BS Newman D 2016 Use of a Surgical Safety Checklist to Improve Team Communication AORN Journal 104(3) 206–216 27568533 10.1016/j.aorn.2016.06.019

[bibr11-17504589221137978] Calland JF Turrentine FE Guerlain S , et al 2011 The surgical safety checklist: Lessons learned during implementation The American Surgeon 77(9) 1131–1137 21944620

[bibr12-17504589221137978] Canadian Patient Safety Institute 2021 TeamSTEPPS Canada Available at https://www.patientsafetyinstitute.ca/en/education/TeamSTEPPS/Pages/default.aspx (accessed April 2022)

[bibr13-17504589221137978] Carney BT West P Neily J Mills PD Bagian JP 2010 Differences in nurse and surgeon perceptions of teamwork: Implications for use of a briefing checklist in the OR AORN Journal 91(6) 722–729 20510945 10.1016/j.aorn.2009.11.066

[bibr14-17504589221137978] Columbus AB Castillo-Angeles M Berry WR Haider AH Salim A Havens JM 2018 An evidence-based intraoperative communication tool for emergency general surgery: A pilot study Journal of Surgery and Research 228 281–289 10.1016/j.jss.2018.03.007 29907223

[bibr15-17504589221137978] Cooke A Smith D Booth A 2012 Beyond PICO: The SPIDER tool for qualitative evidence synthesis Qualitative Health Research 22(10) 1435–1443 22829486 10.1177/1049732312452938

[bibr16-17504589221137978] Covidence 2020 Systematic Review Software Available at www.covidence.org

[bibr17-17504589221137978] D’Amour D Goulet L Labadie JF Martin-Rodriguez LS Pineault R 2008 A model and typology of collaboration between professionals in healthcare organizations BMC Health Services Research 8 188 18803881 10.1186/1472-6963-8-188 PMC2563002

[bibr18-17504589221137978] D’Amour D Oandasan I 2005 Interprofessionality as the field of interprofessional practice and interprofessional education: An emerging concept Journal of Interprofessional Care 19(Suppl 1) 8–20 16096142 10.1080/13561820500081604

[bibr19-17504589221137978] Demetriou C Hu L Smith TO Hing CB 2019 Hawthorne effect on surgical studies ANZ Journal of Surgery 89(12) 1567–1576 31621178 10.1111/ans.15475

[bibr20-17504589221137978] Einav Y Gopher D Kara I , etal 2010 Preoperative briefing in the operating room: shared cognition teamwork and patient safety Chest 137 (2) 443–449 10.1378/chest.08-1732 20133291

[bibr21-17504589221137978] Etherington C Burns JK Kitto S , et al 2021 Barriers and enablers to effective interprofessional teamwork in the operating room: A qualitative study using the Theoretical Domains Framework PLoS ONE 16(4) e0249576 33886580 10.1371/journal.pone.0249576 PMC8061974

[bibr22-17504589221137978] Gillespie BM Gwinner K Chaboyer W Fairweather N 2013 Team communications in surgery – Creating a culture of safety Journal of Interprofessional Care 27(5) 387–393 23672607 10.3109/13561820.2013.784243

[bibr23-17504589221137978] Gore DC Powell JM Baer JG , et al 2010 Crew resource management improved perception of patient safety in the operating room American Journal of Medical Quality 25(1) 60–63 19966113 10.1177/1062860609351236

[bibr24-17504589221137978] Hacquard P Cunat C Toussaint C , et al 2013 [Assessment of the check-list in the operating room: Perceptions of caregivers and physicians (level II assessment)] Annales Françaises d’Anesthèsie et de Rèanimation 32(4) 235–240 10.1016/j.annfar.2013.01.021 23498557

[bibr25-17504589221137978] Halverson AL Andersson JL Anderson K , et al 2009 Surgical team training: The Northwestern Memorial Hospital experience Archives of Surgery 144(2) 107–112 19221320 10.1001/archsurg.2008.545

[bibr26-17504589221137978] Haynes AB Weiser TG Berry WR , et al 2009 A surgical safety checklist to reduce morbidity and mortality in a global population The New England Journal of Medicine 360(5) 491–499 19144931 10.1056/NEJMsa0810119

[bibr27-17504589221137978] Healthcare Excellence Canada 2021 Surgical safety checklist: Canadian version Available at https://www.patientsafetyinstitute.ca/en/toolsResources/Pages/SurgicalSafety-Checklist-Resources.aspx (Accessed April 2022)

[bibr28-17504589221137978] Hinde T Gale T Anderson I Roberts M Sice P 2016 A study to assess the influence of interprofessional point of care simulation training on safety culture in the operating theatre environment of a university teaching hospital Journal of Interprofessional Care 30(2) 251–253 26854195 10.3109/13561820.2015.1084277

[bibr29-17504589221137978] Holmes T Vifladt A Ballangrud R 2020 A qualitative study of how inter-professional teamwork influences perioperative nursing Nursing Open 7(2) 571–580 32089854 10.1002/nop2.422 PMC7024613

[bibr30-17504589221137978] Lingard L Regehr G Orser B , etal 2008 Evaluation of a preoperative checklist and team briefing among surgeons, nurses, and anesthesiologists to reduce failures in communication Archives of surgery 143 (1) 12–17 discussion 18 18209148 10.1001/archsurg.2007.21

[bibr31-17504589221137978] Nundy S Mukherjee A Sexton JB et al 2008 Impact of preoperative briefings on operating room delays: a preliminary report Archives of surgery 143 (11) 1068–1072 19015465 10.1001/archsurg.143.11.1068

[bibr32-17504589221137978] Makary MA Mukherjee A Sexton JB , et al 2007 Operating room briefings and wrong-site surgery Journal of the American College of Surgeons 204 (2) 236–43 17254927 10.1016/j.jamcollsurg.2006.10.018

[bibr33-17504589221137978] Makary MA Holzmueller CG Thompson D , et al 2006 Operating room briefings: Working on the same page Joint Commission Journal on Quality and Patient Safety 32(6) 351–355 16776390 10.1016/s1553-7250(06)32045-4

[bibr34-17504589221137978] Mazzocco K Petitti DB Fong KT , et al 2009 Surgical team behaviors and patient outcomes American Journal of Surgery 197(5) 678–685 18789425 10.1016/j.amjsurg.2008.03.002

[bibr35-17504589221137978] McCulloch P Morgan L New S , et al 2017 Combining systems and teamwork approaches to enhance the effectiveness of safety improvement interventions in surgery: The safer delivery of surgical services (S3) Program Annals of Surgery 265(1) 90–96 28009731 10.1097/SLA.0000000000001589

[bibr36-17504589221137978] Molina G Jiang W Edmondson L , et al 2016 Implementation of the Surgical Safety Checklist in South Carolina Hospitals is associated with improvement in perceived perioperative safety Journal of the American College of Surgeons 222(5) 725–736.e5 10.1016/j.jamcollsurg.2015.12.052 27049781

[bibr37-17504589221137978] Morgan L Hadi M Pickering S , et al 2015 The effect of teamwork training on team performance and clinical outcome in elective orthopaedic surgery: A controlled interrupted time series study BMJ Open 5(4) e006216 10.1136/bmjopen-2014-006216 PMC4410121 25897025

[bibr38-17504589221137978] Muller P Tschan F Keller S , et al 2018 Assessing perceptions of teamwork quality among perioperative team members AORN Journal 108(3) 251–262 30156726 10.1002/aorn.12343

[bibr39-17504589221137978] Munn Z Peters MDJ Stern C Tufanaru C McArthur A Aromataris E 2018 Systematic review or scoping review? Guidance for authors when choosing between a systematic or scoping review approach BMC Medical Research Methodology 18(1) 143 30453902 10.1186/s12874-018-0611-x PMC6245623

[bibr40-17504589221137978] Nguyen VN Miller C Sunderland J McGuiness W 2018 Understanding the Hawthorne effect in wound research-A scoping review International Wound Journal 15(6) 1010–1024 30136375 10.1111/iwj.12968 PMC7949616

[bibr41-17504589221137978] Ong AP Devcich DA Hannam J Lee T Merry AF Mitchell SJ 2016 A ‘paperless’ wall-mounted surgical safety checklist with migrated leadership can improve compliance and team engagement BMJ Quality & Safety 25(12) 971–976 10.1136/bmjqs-2015-004545 26717990

[bibr42-17504589221137978] Reeves S Goldman J Gilbert J , et al 2011 A scoping review to improve conceptual clarity of interprofessional interventions Journal of Interprofessional Care 25(3) 167–174 21182439 10.3109/13561820.2010.529960

[bibr43-17504589221137978] Reeves S Pelone F Harrison R Goldman J Zwarenstein M 2017 Interprofessional collaboration to improve professional practice and healthcare outcomes Cochrane Database of Systematic Reviews 6 CD000072 10.1002/14651858.CD000072.pub3 PMC6481564 28639262

[bibr44-17504589221137978] Reeves S Xyrichis A Zwarenstein M 2018 Teamwork, collaboration, coordination, and networking: Why we need to distinguish between different types of interprofessional practice Journal of Interprofessional Care 32(1) 1–3 29131697 10.1080/13561820.2017.1400150

[bibr45-17504589221137978] San Martin-Rodriguez L Beaulieu MD D’Amour D Ferrada-Videla M 2005 The determinants of successful collaboration: A review of theoretical and empirical studies Journal of Interprofessional Care 19(Suppl 1) 132–147 16096151 10.1080/13561820500082677

[bibr46-17504589221137978] Santana HT Rodrigues MC do Socorro Nantua Evangelista M 2016 Surgical teams’ attitudes and opinions towards the safety of surgical procedures in public hospitals in the Brazilian Federal District BMC Research Notes 9 276 27188751 10.1186/s13104-016-2078-3 PMC4869202

[bibr47-17504589221137978] Schwendimann R Blatter C Dhaini S Simon M Ausserhofer D 2018 The occurrence, types, consequences and preventability of in–hospital adverse events – a scoping review BMC Health Services Research 18(1) 521 29973258 10.1186/s12913-018-3335-z PMC6032777

[bibr48-17504589221137978] Sexton JB Makary MA Tersigni AR , et al 2006 Teamwork in the operating room: Frontline perspectives among hospitals and operating room personnel Anesthesiology 105(5) 877–884 17065879 10.1097/00000542-200611000-00006

[bibr49-17504589221137978] Takala RS Pauniaho SL Kotkansalo A , et al 2011 A pilot study of the implementation of WHO surgical checklist in Finland: Improvements in activities and communication Acta Anaesthesiologica Scandinavica 55(10) 1206–1214 22092125 10.1111/j.1399-6576.2011.02525.x

[bibr50-17504589221137978] Toh LS Lai PSM Othman S Wong KT Low BY Anderson C 2017 An analysis of inter-professional collaboration in osteoporosis screening at a primary care level using the D’Amour model Research in Social & Administrative Pharmacy 13(6) 1142–1150 27780658 10.1016/j.sapharm.2016.10.004

[bibr51-17504589221137978] Tricco AC Lillie E Zarin W , et al 2018 PRISMA Extension for Scoping Reviews (PRISMA-ScR): Checklist and Explanation Annals of Internal Medicine 169(7) 467–473 30178033 10.7326/M18-0850

[bibr52-17504589221137978] Urbach DR Govindarajan A Saskin R Wilton AS Baxter NN 2014 Introduction of surgical safety checklists in Ontario, Canada The New England Journal of Medicine 370(11) 1029–1038 24620866 10.1056/NEJMsa1308261

[bibr53-17504589221137978] Wauben LS Dekker-van Doorn CM van Wijngaarden JD , et al 2011 Discrepant perceptions of communication, teamwork and situation awareness among surgical team members International Journal for Quality in Health Care 23(2) 159–166 21242160 10.1093/intqhc/mzq079 PMC3055275

[bibr54-17504589221137978] World Health Organization (WHO) 2009 Safe surgery saves lives: Guidelines for safe surgery Available at http://apps.who.int/iris/bitstream/handle/10665/44185/9789241598552_eng.pdf?sequence=1 (Accessed April 2022) 23762968

[bibr55-17504589221137978] World Health Organization (WHO) 2010 Framework for action on interprofessional education and collaborative practice Available at https://apps.who.int/iris/handle/10665/70185 (Accessed April 2022) 21174039

[bibr56-17504589221137978] World Health Organization (WHO) 2021 Draft global patient safety action plan 2021-2030 Available at https://cdn.who.int/media/docs/default-source/patient-safety/gpsap/final-draft-global-patient-safety-action-plan-2021-2030.pdf?sfvrsn=fc8252c5_5 (Accessed April 2022)

[bibr57-17504589221137978] Zegers M de Bruijne MC de Keizer B , et al 2011 The incidence, root-causes, and outcomes of adverse events in surgical units: Implication for potential prevention strategies Patient Safety in Surgery 5 13 21599915 10.1186/1754-9493-5-13 PMC3127749

[bibr58-17504589221137978] Zwarenstein M Goldman J Reeves S 2009 Interprofessional collaboration: Effects of practice-based interventions on professional practice and healthcare outcomes Cochrane Database of Systematic Reviews 3 CD000072 10.1002/14651858.CD000072.pub2 19588316

