# Supplementary material for: Interventions to facilitate interprofessional collaboration in the operating theatre: A scoping review
Source: J Perioper Pract. 2022 Dec 5;34(1-2):6–19. doi: 10.1177/17504589221137978 (PMC10771025; doi:10.1177/17504589221137978)
Supplement: sj-docx-1-ppj-10.1177_17504589221137978 – Supplemental material for Interventions to facilitate interprofessional collaboration in the operating theatre: A scoping review [file sj-docx-1-ppj-10.1177_17504589221137978.docx]

**Appendix A**

**Scoping Review Searches**

**OVID MEDLINE**

Interprofessional Collaboration in the OR: A Nursing Perspective

Octobre 31, 2020

1. Perioperative Nursing/ or Operating Room Nursing/ or Nursing/ or Postanesthesia Nursing/

2. Nurses/ or Nurse's Role/

3. nurs*.ti,ab,kw.

4. 1 or 2 or 3

5. interprofessional relations/ or interdisciplinary communication/ or physician-nurse relations/

6. Patient Care Team/

7. (interprofessional* adj3 collaborat*).ti,ab,kw.

8. (interprofessional* adj3 relation*).ti,ab,kw.

9. (interdisciplinary adj3 collaborat*).ti,ab,kw.

10. (multidisciplinary adj3 collaborat*).ti,ab,kw.

11. 5 or 6 or 7 or 8 or 9 or 10

12. Operating Rooms/

13. perioperative care/ or intraoperative care/ or perioperative nursing/ or postoperative care/

14. (operating adj2 room*). ti,ab,kw.

15. (operating adj2 theat*).ti,ab,kw.

16. perioperative.ti,ab,kw.

17. 12 or 13 or 14 or 15 or 16

18. 4 and 11 and 17

19. limit 18 to yr="2005 -Current" (**results:** **732**)

**OVID PsycINFO**

Interprofessional Collaboration in the OR: A Nursing Perspective

Novembre 6, 2020

1. nurses/ or nursing/

2. Roles/ or Health Personnel Attitudes/

3. nurs*.ti,ab.

4. 1 or 2 or 3

5. Health Personnel/ or Interdisciplinary Treatment Approach/ or Health Care Services/ or Teamwork/ or Collaboration/

6. Interpersonal Communication/

7. (interprofessional* adj3 collaborat*).ti,ab.

8. (interprofessional* adj3 relation*).ti,ab.

9. (interdisciplinary adj3 collaborat*).ti,ab.

10. (multidisciplinary adj3 collaborat*).ti,ab.

11. 5 or 6 or 7 or 8 or 9 or 10

12. surgery/ or postsurgical complications/ or surgical patients/

13. (operating adj2 theat*).ti,ab.

14. (operating adj2 room*).ti,ab.

15. perioperative.ti,ab.

16. 12 or 13 or 14 or 15

17. 4 and 11 and 16

18. limit 17 to yr="2005 -Current" **(results: 118)**

**CINAHL**

Interprofessional Collaboration in the OR: A Nursing Perspective

Novembre 6, 2020

1. (MH "Perioperative Nursing")

2. (MH "Perianesthesia Nursing")

3. (MH "Nursing Role")

4. (MH "Nurses+")

5. "nurs*"

6. S1 OR S2 OR S3 OR S4 OR S5

7. (MH "Interpersonal Relations") OR (MH "Interprofessional Relations") OR (MH "Intraprofessional Relations") OR (MH "Nurse-Patient Relations") OR (MH "Nurse-Physician Relations")

8. (MH "Multidisciplinary Care Team")

9. (interprofessional* N3 collaborat*)

10. (interprofessional* N3 relation*)

11. (interdisciplinary N3 collaborat*)

12. (multidisciplinary N3 collaborat*)

13. S7 OR S8 OR S9 OR S10 OR S11 OR S12

14. (MH "Operating Rooms")

15. (MH "Perioperative Care") OR (MH "Intraoperative Care") OR (MH "Postoperative Care") OR (MH "Preoperative Care") OR (MH "Postoperative Period") OR (MH "Preoperative Period")

16. (operating N2 room*)

17. (operating N2 theat*)

18. perioperative or intraoperative

19. S14 OR S15 OR S16 OR S17 OR S18

20. S6 AND S13 AND S19

21. S6 AND S13 AND S19 Limiters - Published Date: 20050101-20201231 **(results: 1,259)**

**OVID EMBASE**

Interprofessional Collaboration in the OR: A Nursing Perspective

17 Novembre 2020

1. Perioperative Nursing/ or Operating Room Nursing/ or Nursing/ or Postanesthesia Nursing/

2. Nurses/ or Nurse's Role/

3. nurs*.ti,ab,kw.

4. 1 or 2 or 3

5. Patient Care Team/

6. interdisciplinary communication/

7. doctor nurse relation/

8. patient care/

9. (interprofessional* adj3 collaborat*).ti,ab,kw.

10. (interprofessional* adj3 relation*).ti,ab,kw.

11. (interdisciplinary adj3 collaborat*).ti,ab,kw.

12. (multidisciplinary adj3 collaborat*).ti,ab,kw.

13. 5 or 6 or 7 or 8 or 9 or 10 or 11 or 12

14. operating room/

15. perioperative period/

16. (operating adj2 room*).ti,ab,kw.

17. (operating adj2 theat*).ti,ab,kw.

18. perioperative.ti,ab,kw.

19. 14 or 15 or 16 or 17 or 18

20. 4 and 13 and 19

21. limit 20 to yr="2005 -Current" **(results: 424)**
